# A beamline to control longitudinal phase space whilst transporting laser wakefield accelerated electrons to an undulator

**DOI:** 10.1038/s41598-023-35435-7

**Published:** 2023-05-31

**Authors:** Kay A. Dewhurst, Bruno D. Muratori, Enrico Brunetti, Bas van der Geer, Marieke de Loos, Hywel L. Owen, S. Mark Wiggins, Dino A. Jaroszynski

**Affiliations:** 1 Department of Physics and Astronomy, The University of Manchester, Manchester, M13 9PL UK; 2 The Cockcroft Institute, Warrington, WA4 4AD UK; 3 ASTeC, UKRI-STFC Daresbury Laboratory, Warrington, WA4 4FS UK; 4 SUPA, Department of Physics, University of Strathclyde, Glasgow, G4 0NG UK; 5 Pulsar Physics, Eindhoven, 5614 BC The Netherlands; 6 Present Address: Beams Department (BE), CERN, 1211 Geneva, Switzerland

**Keywords:** Experimental particle physics, Plasma-based accelerators

## Abstract

Laser wakefield accelerators (LWFAs) can produce high-energy electron bunches in short distances. Successfully coupling these sources with undulators has the potential to form an LWFA-driven free-electron laser (FEL), providing high-intensity short-wavelength radiation. Electron bunches produced from LWFAs have a correlated distribution in longitudinal phase space: a chirp. However, both LWFAs and FELs have strict parameter requirements. The bunch chirp created using ideal LWFA parameters may not suit the FEL; for example, a chirp can reduce the high peak current required for free-electron lasing. We, therefore, design a flexible beamline that can accept either positively or negatively chirped LWFA bunches and adjust the chirp during transport to an undulator. We have used the accelerator design program MAD8 to design a beamline in stages, and to track particle bunches. The final beamline design can produce ambidirectional values of longitudinal dispersion ($R_{56}$): we demonstrate values of + 0.20 mm, 0.00  mm and − 0.22 mm. Positive or negative values of $R_{56}$ apply a shear forward or backward in the longitudinal phase space of the electron bunch, which provides control of the bunch chirp. This chirp control during the bunch transport gives an additional free parameter and marks a new approach to matching future LWFA-driven FELs.

## Introduction

Laser wakefield accelerators (LWFAs) have been developed over recent decades and can now produce electron bunches with energies above 1 GeV^1–4^, small normalised transverse emittances of less than 1 $\upmu \text {m}$^5–7^ and short bunch durations of 1 fs to 10  fs^8–12^. The short bunch duration allows a modest bunch charge, for example around 50 pC, to result in a high peak current $>{1}\,\text {pA}$. These three properties of high energy, low emittance and high peak current make LWFA electron sources appealing as drivers of free-electron lasers (FELs) in the ultraviolet to soft x-ray spectral range. LWFAs have been recognised as excellent potential drivers of FELs since 2002^13^. The high accelerating gradients sustained in the plasma enable LWFAs to accelerate electrons to GeV-scale energies in short millimetre- to centimetre-scale distances. Linking a compact, GeV-energy, LWFA electron source to an undulator could reduce the size of a free-electron laser from a device hosted at a national facility to one installed at individual university or industry sites. This would increase access to FEL radiation, which is now widely used for time-resolved, nano-scale resolution studies of samples in many fields, including chemistry, biology and materials science^14^.

Although LWFA sources have produced electron bunches with measured properties of up to 7.8 GeV energy^4^, as low as 0.056 $\upmu \text {m}$ normalised transverse emittance^7^ and as short as 1.4 fs duration^9^, these parameters have not yet been achieved together and generally degrade as the bunch travels out of the plasma and downstream in the accelerator system. The bunches from LWFA sources have characteristically large relative energy spread (around 1%) and divergence (around 1 mrad) compared with conventional sources. The large energy spread and divergence can lead to a degradation of favourable bunch properties before reaching an undulator: for example, causing the normalised transverse emittance to grow significantly with distance from the source^15–19^. To improve the bunch properties at the undulator entrance two approaches can be taken^17^: (i) further improving the bunch properties at the electron source by adjusting the plasma e.g. gas type(s)^20,21^, gas density profile^22,23^ and laser pulse profile^22,24^, or (ii) ameliorating some of the less-favourable bunch properties during the transport process between the source and the undulator. In this paper, we use the latter approach to design a beamline that can adjust the length of a bunch with some initial chirp.

**Figure 1:**
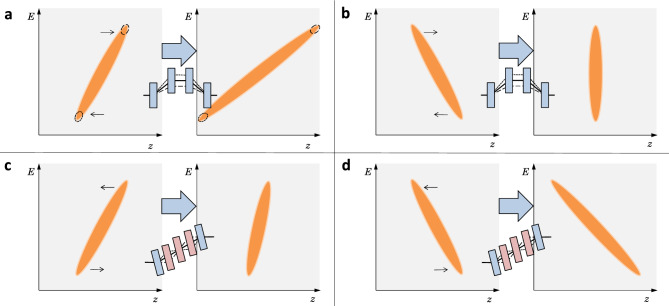
Illustrations to show the effect of chicanes (top) and doglegs (bottom) on the longitudinal phase space profiles of chirped bunches (orange). Plots show energy *E* against position *z*. In a chicane, (**a**) and (**b**), higher energy particles take a shorter path (dash) through the magnets than lower energy particles (dot) causing the bunch to shear forward i.e. the high energy particles move forward with respect to the bunch centre. A bunch with an initial positive chirp, as in (**a**) and (**c**), is lengthened by a chicane (**a**) or shortened by a dogleg (**c**). A bunch with an initial negative chirp, as in (**b**) and (**d**), is shortened by a chicane (**b**) or lengthened by a dogleg (**d**). As the magnitude of $R_{56}$ is typically larger in a chicane than in a dogleg, a chicane will have a more pronounced effect on the bunch chirp.

LWFA electron sources typically produce bunches that are chirped^12,25^; the particle energy is correlated with position along the bunch length. In this paper, we define a bunch with a positive chirp as having a positive slope on an energy-versus-position (bunch length) longitudinal phase-space plot i.e. higher energy particles arrive at an earlier time. An example of a bunch with a positive chirp is shown in Fig. 1a. Conversely, a bunch with a negative chirp would have a negative slope. There is some disagreement in the literature, based on simulations, as to whether the chirp from an LWFA electron source is expected to be negative^24–27^ or positive^28,29^ and this may depend on the particular LWFA configuration. One experiment confirmed that the electron bunch chirp from an LWFA source can be linear^12^, but this experiment was not designed to determine the chirp orientation. The beamline we present here can accommodate both cases; transporting bunches with either initially positive or negative chirps whilst adjusting the chirp during transport. In addition to improving matching between the LWFA and undulator, this beamline can act as a diagnostic for the LWFA; using a known adjustment in chirp would allow the initial bunch chirp to be determined experimentally for the first time.

Previous experiments linking LWFA sources to undulators used simple beamlines of only a few quadrupoles to lessen the divergence of the electron bunch before it entered the undulator. These experiments successfully demonstrated the production of incoherent radiation at visible^30^, ultraviolet^31,32^ and soft x-ray^33^ wavelengths. To increase the brightness of these sources more sophisticated beamlines are being designed and built at facilities around the world, with the first LWFA-FEL proof-of-principle radiation demonstrated in 2021^34^. These beamline designs broadly fall into two categories based on how they account for the large energy spread of the LWFA electron bunches. The first uses a dipole or dogleg to introduce transverse dispersion to the electron beam and matches this into a transverse gradient undulator (TGU)^35,36^. The TGU is designed with canted magnet blocks so there is a transverse profile in the magnetic field strength, allowing different energy parts of the bunch to emit radiation at the same wavelength^37^. The second type utilises longitudinal dispersion, introduced by a magnetic chicane, to stretch the bunch longitudinally^38,39^. This approach reduces the transverse emittance of each coherent slice of the bunch, promoting the production of FEL radiation^40,41^. The beamline presented here falls into the second category as we focus on adjusting the bunch length by varying the longitudinal dispersion ($R_{56}$). As this beamline can adjust the chirp in either direction, it provides additional flexibility to previous beamlines in this category.

Longitudinal dispersion ($R_{56}$) can be adjusted in a beamline using magnetic elements such as chicanes, arcs and doglegs. At relativistic energies, electrons in a bunch will not significantly outrun each other due to velocity differences (ballistic lengthening). For example, at 1 GeV an electron bunch would only ballistically lengthen by $1/{\gamma }^2={0.26}\,\upmu \text {m}$ per meter of transport. Magnetic elements can change the longitudinal position of electrons in a bunch based on their energy by introducing path length differences for electrons with different energies. For example, Fig. 1a shows the longer path taken by lower energy electrons (dotted line) and the shorter path taken by higher energy electrons (dashed line) through a four-dipole chicane. The effect of these path length differences is quantified by the value of longitudinal dispersion ($R_{56}$). Magnetic chicanes produce a positive or chicane-like $R_{56}$ value, imparting a shear onto an electron bunch travelling through the chicane so that high-energy particles move ahead of low-energy particles; this appears as a forward shear on a longitudinal phase-space plot of energy versus position. Conversely, doglegs naturally produce an $R_{56}$ value of the opposite sign (negative), which is described in this paper as dogleg-like $R_{56}$. Consequently, a dogleg imparts a shear onto the electron bunch in the opposite direction to a chicane, as shown in Fig. 1. By combining features of a dogleg and a chicane, our beamline can produce both chicane-like and dogleg-like values of $R_{56}$, as well as a neutral value of $R_{56} = 0$. This allows for an initially chirped bunch to be lengthened, shortened or transported without a dispersion effect.

A unique feature of this beamline is its ability to adjust the orientation (chirp) of an LWFA electron bunch in longitudinal phase space by imparting either a forward or backward shear to the bunch. This allows bunches with initial chirps that are either positive or negative to be adjusted to support the production of free-electron laser (FEL) radiation in a downstream undulator. Therefore, this beamline provides a flexible system suitable for a variety of experiments linking an LWFA electron source with an undulator and can contribute to the design of a future compact LWFA-driven FEL.

## Methods

**Figure 2:**
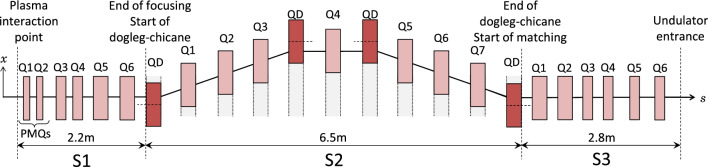
The layout of the full beamline (top view). The focusing section (S1) comprises two adjustable-strength permanent magnet quadrupoles (S1Q1 and S1Q2) followed by four electromagnetic quadrupoles. The main dogleg-chicane section (S2) comprises eleven electromagnetic quadrupoles, each on an individual translation stage so they can be offset in the *x*-direction. The undulator matching section (S3) comprises six electromagnetic quadrupoles. The darker shaded quadrupoles, labelled QD, are offset from the reference trajectory (magnet centre indicated with dashed line) to bend the beam. Quadrupoles S1Q1 and S1Q2 have a magnetic length of 100 mm, quadrupoles S1Q3, S1Q4, S3Q3, S3Q4, S3Q5 and S3Q6 have a magnetic length of 150 mm and all the other quadrupoles have a magnetic length of 250 mm.

The beamline is designed in three sections as shown in Fig. 2. First is the focusing section (S1), which reduces the large divergence of the initial LWFA particle bunch. The second section (S2) is the main hybrid dogleg-chicane design, which can be adjusted to vary the bunch chirp. The third section (S3) matches the beam from the second section into an undulator. Reasonable start and end conditions must be known for successful bunch transport. The start conditions (the initial bunch parameters) are based on measurements of currently attainable laser wakefield accelerated bunches. The end conditions come from the parameters of the undulator. The beamline is designed for installation at Strathclyde University’s SCAPA (Scottish Centre for the Application of Plasma-based Accelerators) facility^42^, so we choose the available on-site ALPHA-X undulator^43^ as an example for this study. The ALPHA-X undulator has a suitable period $\lambda _{u}$ for producing the desired soft x-ray (2.93  nm) radiation with the expected 1.0 GeV energy electrons. This choice of undulator and location limits the length of the beamline to 13.9 m from the plasma exit to the undulator entrance. A summary of the methods is presented below; further details are available in the reference^44^.

### Start and end conditions

#### Initial bunch parameters

The beamline is designed to transport laser wakefield accelerated electron bunches with a central energy of 1.0 GeV. A selection of high-quality electron bunches previously produced from laser wakefield accelerators and measured with energies around 1 GeV are summarised in Table 1. The parameters in Table 1 show that a bunch with 1 GeV central energy, 1% relative energy spread, 1 mrad divergence and 50 pC charge is representative of a currently attainable electron bunch from an LWFA. We use these realistic parameters to create an initial bunch distribution for our simulations.

**Table 1:** A selection of experimentally measured electron bunch parameters with energies around 1 GeV.

Laser wakefield acceleration scheme	Gas [gas 1][gas 2]	Central energy/GeV	Energy spread/%	Divergence/mrad	Charge/pC
Capillary discharge^1^	[H_2_]	1.0*	$\le 2.5$	1.6	30
Gas cell^2^	[He]	0.95	7 (11)	0.5	13
Gas cell^2^	[He]	2.0	6 (10)	0.6	63
Cascaded gas jets^26^	[He][He]	0.58	1	0.2	10–70
Cascaded gas jets^45^	[He][He]	0.42	1.4	$\le 0.5$	46
Cascaded gas cells^46^	[He+N_2_][He]	0.46	5	2.3	35

These parameters represent what is achievable from current laser wakefield acceleration sources including gas jets, gas cells and electrical-discharge capillaries. Cascaded refers to gas targets in series. Two values of energy spread are given where two methods were used to determine this parameter, with the more conservative value in parentheses. The charge reported is the single bunch charge. In some cases (*) the reported bunch is produced alongside a secondary lower-energy bunch.

The duration of LWFA electron bunches has been measured to be only a few femtoseconds^8–11^ and simulations predict attosecond bunches^11,47^ may soon be possible, therefore, we choose a bunch duration of 1 fs as a likely parameter. The transverse size of the electron bunch increases as it exits the plasma; the root-mean-squared (r.m.s.) transverse bunch size at the plasma exit has been measured in different LWFA experiments to be 0.9 $\upmu \text {m}$^5^, 4 $\upmu \text {m}$^48^ and 6 $\upmu \text {m}$^49^. We select an initial transverse beam size of 5 μm as a currently attainable parameter. By assuming a beam waist at the plasma exit, the geometric emittance $\epsilon _{x}$ can be estimated from the product of the transverse beam size $\sigma _{x}$ and divergence $\sigma _{x'}$; $\epsilon _{x}=\sqrt{\langle {x}^2\rangle \langle {p_x}^2\rangle -\langle {x}{p_x}\rangle ^2}=\sqrt{\langle {x}^2\rangle \langle {p_x}^2\rangle -0}\approx \sigma _{x}\sigma _{x'}$. Using our chosen parameters of 1 mrad divergence and 5 $\upmu \text {m}$ transverse source size, the initial bunch has a geometric emittance of $5 \times 10^{-3}\,\upmu \text {m}$ and a normalised emittance of 9.8 $\upmu \text {m}$. A summary of all these initial bunch parameters is presented in Table 2, including the derived initial Twiss parameters $\alpha _{x,y}$ and $\beta _{x,y}$ used in the beamline matching process.

**Table 2:** Start conditions.

Parameter	Value	Unit
Central energy	1.0	GeV
Lorentz factor $\gamma _{0}$	1958	
Energy spread (rms)	1	%
Transverse size (rms)	5	$\upmu \text {m}$
Divergence (rms)	1	mrad
Normalised emittance	9.79	$\upmu \text {m}$
Geometric emittance	$5 \times 10^{-3}$	$\upmu \text {m}$
Charge	50	pC
Bunch length (rms)	1 (0.3)	fs ($\upmu \text {m}$)
Twiss $\alpha _{x,y}$	0, 0	
Twiss $\beta _{x,y}$	5, 5	mm

Properties of the input bunch distributions used in this study. The Twiss parameters $\alpha _{x,y}$ and $\beta _{x,y}$ provide the start conditions for matching in MAD8.

#### Undulator parameters

The beamline design incorporates a 1.5 m long undulator, one of two previously used in the ALPHA-X project^43^. The parameters of this undulator are given in Table 3. The undulator gap height can be manually adjusted to vary the deflection parameter *K*; for this study, the minimum gap height of 3.5 mm was chosen to give the maximum electron deflection ($K=1.0$). Electrons passing through an undulator oscillate, producing radiation with a fundamental wavelength ($\lambda _{r}$) given by the undulator equation:

1 $$\begin{aligned} \lambda _{r}=\frac{\lambda _{u}}{2\gamma _{0}^{2}}\left( 1+\frac{K^{2}}{2} \right) , \end{aligned}$$

where $\lambda _{u}$ is the period length of the undulator dipole magnets and $\gamma _{0}$ is the Lorentz factor associated with the electron energy. Therefore 1.0 GeV electrons will produce soft x-ray radiation ($\lambda _{r}={2.93}\,\text {nm}$) when traversing the ALPHA-X undulator. The undulator is modelled as an array of dipoles in the accelerator design programme MAD8^50^. The Twiss parameters at the entrance of the model undulator are varied until a symmetric solution is found that focuses the electron beam at the undulator centre. These resultant Twiss parameters are included in Table 3 and provide the end conditions for matching the beamline.

**Table 3:** End conditions.

Parameter	Value	Unit
Undulator length	1.5	m
Number of periods $N_{u}$	100	
Period length	15	mm
Operating gap	3.5	mm
Deflection parameter *K*	1.0	
Twiss $\alpha _{x,y}$	0.45, 1.00	
Twiss $\beta _{x,y}$	2.01, 1.49	mm

Parameters of the ALPHA-X undulator^43^ used in this study.

The Twiss parameters $\alpha _{x,y}$ and $\beta _{x,y}$ were found from a MAD8 model of the undulator and provide the end conditions for matching in MAD8.

### Beamline design

#### Focusing section (S1)

The focusing section (S1) is designed to match the initial Twiss parameters of the input bunch to the parameters required for the main dogleg-chicane section (S2); a key feature is control of the beam divergence. Laser wakefield accelerated electron bunches are divergence-dominated, which leads to a growth of the normalised transverse emittance in the drift space following the plasma exit^15–18^. The divergence can be controlled with strong focusing and the emittance growth can be minimised by reducing the drift length. Permanent magnet quadrupoles (PMQs) can achieve strong focusing close to the plasma exit and have been successfully implemented in previous LWFA experiments^30,32,33,51^. PMQs are more compact than electromagnets and can be made vacuum compatible, allowing them to be located close to the plasma source, within the same vacuum chamber.

**Figure 3:**
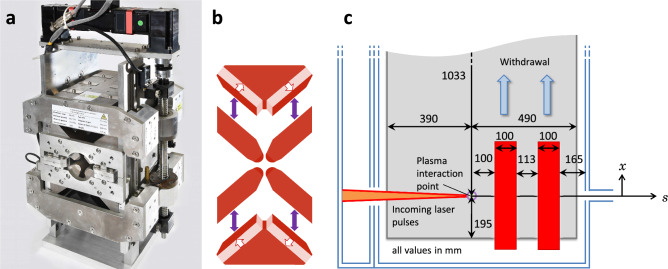
ZEPTO quadrupole^52^: an adjustable-strength permanent magnet. (**a**) A photo of the prototype high gradient ZEPTO quadrupole^53^. Photograph courtesy of STFC. (**b**) The pole movement of the ZEPTO quadrupole. The central aperture is made of four fixed-position steel pieces. The magnetic material can be moved in to give the maximum field gradient or moved out to reduce the field gradient; the direction of movement is indicated by purple arrows. White arrows point along the easy axis of the magnetic material. (**c**) The layout of the vacuum chamber (top view) with lengths given in millimetres. Two adjustable strength ZEPTO quadrupoles (red) are located in the same vacuum chamber as the LWFA source (plasma interaction point). The quadrupoles can be withdrawn to another area of the vacuum chamber using translation stages when they are not required for an experiment.

The focusing section must be adjustable to match the LWFA bunch to different set-ups of the dogleg-chicane section (S2). Therefore, we incorporate PMQs which have movable magnetic material that allows the strength to be adjusted without changing the position of the PMQ or the aperture size. We choose an adapted version of the ZEPTO^52^ quadrupole design with an inscribed diameter of 6 mm, field gradient range of ${25}\,\text {Tm}^{-1}$ to ${100}\,\text {Tm}^{-1}$ and magnetic length of 100 mm. Adding a metallic seal coat^54^ to the magnetic material and using vacuum grease on the movable parts will make the ZEPTO quadrupole design vacuum compatible. Two of these magnets can be located inside the vacuum chamber close to the plasma source, as shown in Fig. 3. The focusing section comprises six magnets; two in-vacuum adjustable-strength PMQs, followed by four electromagnetic quadrupoles. The electromagnetic quadrupoles have an inscribed diameter of 21 mm and a maximum field gradient of ${75}\,\text {Tm}^{-1}$. The layout of the focusing section (S1) is shown in Fig. 2.

First, the dogleg-chicane section (S2) was modelled to give a longitudinal dispersion value of $R_{56}={0}\,\text {mm}$, which corresponds to no change in bunch chirp; then, the focusing section (S1) was set up to match into S2. The drift lengths and magnet strengths in the focusing section (S1) were varied in pairs using MAD8’s inbuilt downhill-simplex method until the Twiss parameters matched with section S2 and the maximum value of the beta-functions were reduced as far as possible. The magnet positions for the focusing section were then fixed and only the strengths were adjusted to match Twiss parameters with the other (chirp adjustment) set-ups of the dogleg-chicane (S2). This avoids changing any of the magnet positions between different set-ups of the beamline.

#### Dogleg-chicane section (S2)

**Figure 4:**
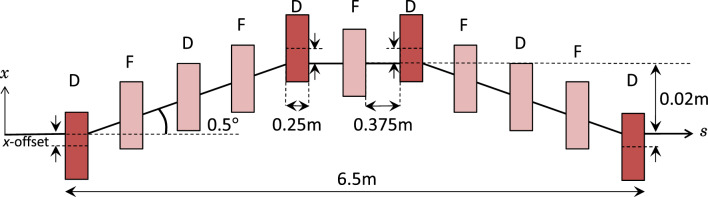
The layout of the dogleg-chicane section (S2) (top view) comprises five horizontally focusing (F) and six defocusing (D) quadrupoles (red). Each quadrupole has a magnetic length of 0.25 m and is separated by a drift space of 0.375 m. Four of the quadrupoles (dark red) have an *x*-offset to bend the electron trajectory by ${0.5}^{\circ }$. The amount of *x*-offset is determined in the beamline matching process to be 4.1 mm. Considering the four bending quadrupoles (darker red), the design resembles a chicane. The first five quadrupoles resemble a dogleg, as do the final set of five quadrupoles. Therefore this quadrupole channel combines design features of both chicanes and doglegs.

The main dogleg-chicane section of the design aims to provide chirp adjustment by producing both dogleg-like (negative) and chicane-like (positive) values of $R_{56}$. The layout of this section is shown in Fig. 4. The design comprises eleven quadrupoles; four of these are offset from the reference trajectory to act as combined-function magnets. Offset quadrupoles were chosen as they both bend and focus the electron bunch; a principle previously demonstrated in the fixed-field circular accelerator EMMA^55^, whose ring was completely composed of offset quadrupoles. Using offset quadrupoles mounted on translation stages, as shown in Fig. 2, adds the flexibility to transform section S2 into a straight beamline if required by experiment, for example, to benchmark the $R_{56}={0}\,\text{mm}$ case. In the designed layout (shown in Fig. 4), the four offset quadrupoles bend the electron trajectory away from and back onto a straight line; resembling the layout of a chicane. Between the first and final pairs of offset quadrupoles are three on-axis quadrupoles; resembling a dogleg. The layout of this section (S2), therefore combines elements of both conventional doglegs and chicanes.

A small bend angle of ${0.5}^{\circ }$ is chosen to minimise the production of coherent synchrotron radiation by the short electron bunches, which may degrade the bunch emittance^56^. However, the ${0.5}^{\circ }$ angle is large enough to separate the electrons from any remaining laser light or radiation produced within the plasma, which can be detected downstream as a diagnostic for the LWFA process. The first two offset quadrupoles are separated by 2.5 m, which results in an offset of the electron beam of around 0.02 m from the straight-through position, as illustrated in Fig. 4.

The dogleg-chicane was modelled as a symmetric system in MAD8 using seven quadrupoles to represent the on-axis quadrupoles and four combined function magnets to represent the offset quadrupoles (darker red in Fig. 4). Modelling the offset quadrupoles as combined function magnets allowed the dipole and quadrupole field strengths to be varied independently. A set-up for this section (S2), which could achieve an $R_{56}$ value of 0 mm was initially considered, before set-ups with maximum and minimum values of $R_{56}$ were explored.

To find a set-up with $R_{56}={0}\,\text{mm}$, a single dogleg section (the first five quadrupoles) was modelled and the magnet strengths were adjusted to close the dispersion. MAD8 did not converge on a solution for the small bend angle of ${0.5}^{\circ }$ in a single iteration, so a larger angle (e.g. ${30}^{\circ }$) dogleg was simulated in the first iteration, which allowed MAD8 to converge on a solution for the ${0.5}^{\circ }$ case in a second iteration. Two dogleg sections were then placed together (the first and final five quadrupoles) with a central drift space to form a dogleg-chicane. The magnet strengths were re-adjusted to close the dispersion at the beamline end. The on-axis quadrupoles were adjusted in pairs to give a symmetric solution and the four offset quadrupoles were varied together so they would have equal strengths and offsets in the final design. Finally, the central quadrupole was added allowing the line to resemble a FODO channel; the strength of this central quadrupole was varied independently. The parameter search for an $R_{56}={0}\,\text{mm}$ solution was carried out in two steps: first, to give $R_{56}={0}\,\text{mm}$ and zero dispersion at the beamline-end, and second, to give small values of the beta-functions. These two steps were carried out three times until MAD8 converged on a symmetric solution that met all three criteria: zero $R_{56}$, closed dispersion and reasonable beta-function ($\beta _{x,y}<{100}\,\text {m}$). This solution, with $R_{56}={0}\,\text{mm}$, is considered a neutral solution as the effect of the dogleg-chicane does not cause bunch lengthening. However, other lengthening effects, for example, divergence-driven lengthening, where more divergent particles take an outer (longer) path through the quadrupoles, are still experienced by the bunch.

After the set-up with $R_{56}={0}\,\text{mm}$ was found, searches were carried out to find set-ups with the maximum chicane-like and maximum dogleg-like values of $R_{56}$. To prevent movement of the offset quadrupoles between set-ups, their strengths were not varied during these searches. To find the maximum chicane-like value of $R_{56}$, positive values of $R_{56}$ from 0.1 to 0.5 mm were set as a matching constraint within MAD8 and the quadrupole strengths varied to find a solution. Each search was carried out using MAD8’s inbuilt downhill-simplex algorithm. Each solution was checked for the value of $R_{56}$ achieved, the quadrupole strengths required and the maximum values of the beta-functions. The best solution was considered to have the largest achieved value of $R_{56}$, balanced against reasonable quadrupole strengths ($|k|\lesssim {20}\,\text {m}^{-2}$) and beta-functions ($\beta _{x,y}<{100}\,\text {m}$). The same approach was used to find a set-up with the maximum dogleg-like value of $R_{56}$. In this case, negative values of $R_{56}$ from − 0.1 to − 0.25 mm were set as a matching constraint. As before, the best solution was considered to have the largest magnitude achieved value of $R_{56}$ balanced against reasonable quadrupole strength and beta-function values.

#### Undulator matching section (S3)

The undulator matching section (S3) is designed to match the Twiss parameters at the end of the dogleg-chicane section (S2) to those at the undulator entrance (the end conditions). Three set-ups of the dogleg-chicane (S2) were achieved (cases of zero, chicane-like and dogleg-like $R_{56}$) then, the focusing section (S1) was added and adjusted for each of these set-ups. Finally, each set-up was matched into the undulator by the undulator matching section (S3). This third section of the beamline (S3) comprises six electromagnetic quadrupoles as shown in Fig. 2: the first two quadrupoles have a magnetic length of 250 mm and the final four are 150 mm. The initial drift lengths used were 0.2 m for the first four drifts, followed by two drift lengths of 0.3 m.

This section (S3) was adjusted in a similar way to the focusing section (S1); the $R_{56}={0}\,\text{mm}$ set-up was considered first. The magnet strengths were adjusted in pairs to reduce the Twiss parameters at the beamline end. The quadrupoles were not constrained to a FODO pattern; this is in contrast to the focusing section (S1), as the PMQs in S1 are not easily rearranged from horizontally to vertically focusing. The MAD8 model of the undulator was then added and the final four quadrupole strengths were re-adjusted to give small beta-functions ($\beta _{x,y}<{5}\,\text {m}$) throughout the length of the 1.5 m undulator: ensuring a beam waist in both *x* and *y* occurs close to the undulator centre. This process was repeated for the chicane-like and dogleg-like $R_{56}$ set-ups of the beamline. Solutions for all three beamline set-ups were found without the need to adjust the drift lengths, which allows all magnets to remain in fixed positions.

### Particle tracking

Particle bunches were tracked through the three beamline set-ups with linear optics to demonstrate their effect on the bunch chirp. Input bunches consisting of 10,000 macro-particles with r.m.s. bunch parameters from Table 2 were created using the ASTRA generator program^57^. Three bunches were created with either no chirp, negative chirp or positive chirp. For the chirped bunches the slice energy spread was set to 0.1% so that the overall relative energy spread of the bunch remained consistent with Table 2 at 1%. The bunch distributions were converted for input to the MAD8 tracking module^50^ which was used to track the particle distributions through each set-up of the final beamline design.

## Results

### Beamline configurations

#### Dogleg chicane section (S2)

**Figure 5:**
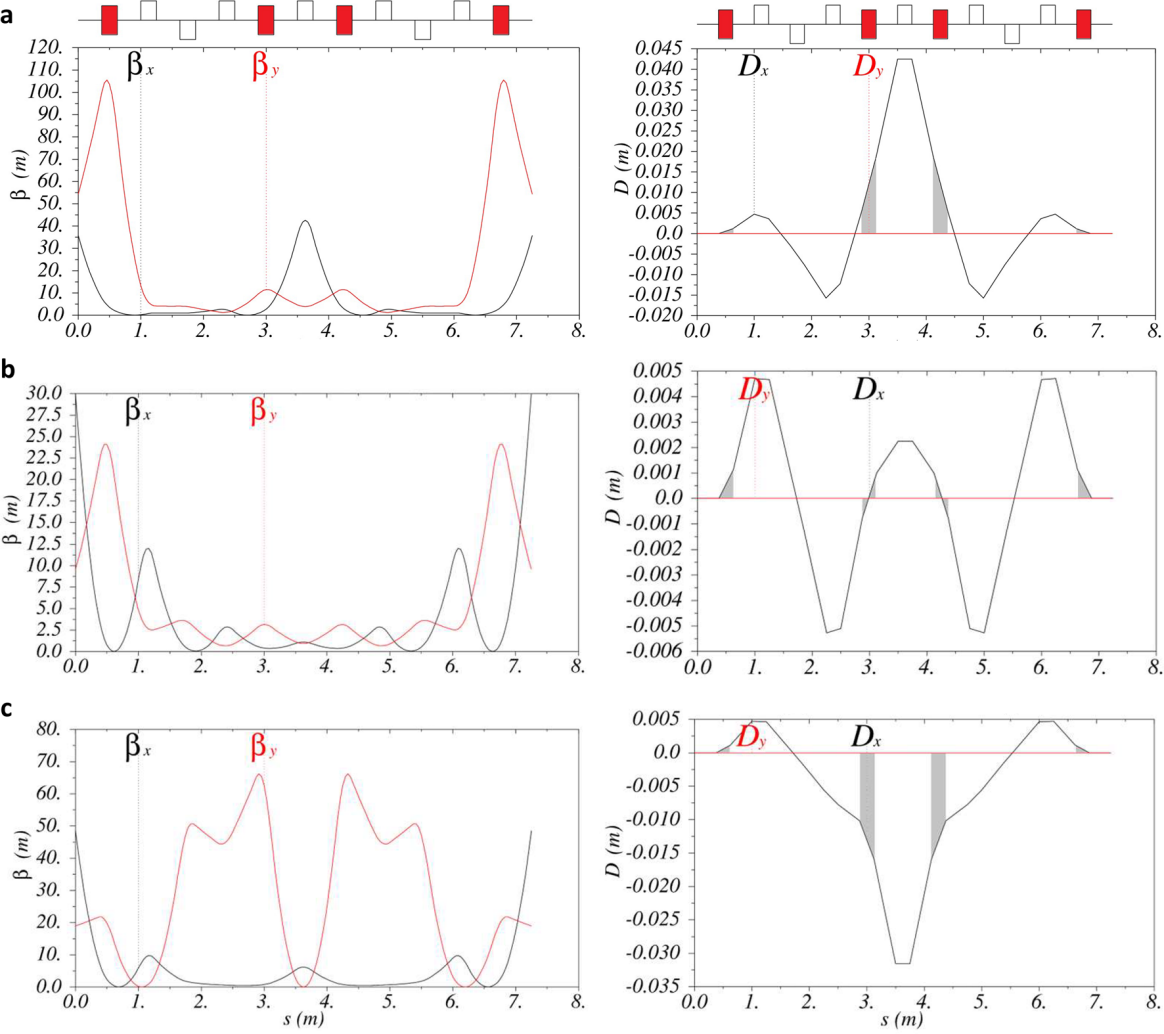
Beta-functions (left panels) and dispersion functions (right panels) in the dogleg-chicane section (S2) for the three set-ups of $R_{56}$. (**a**) Plots for the chicane-like case with the maximum chicane-like value achieved of $R_{56}={0.20}\,\text{mm}$. (**b**) Plots for a neutral solution with $R_{56}={0.00}\,\text{mm}$. (**c**) Plots for the dogleg-like case with the maximum dogleg-like value achieved of $R_{56}={-0.22}\,\text{mm}$. The quadrupole layout is shown above the plots indicating on-axis (empty rectangles) and off-axis (filled rectangles) quadrupoles. The (grey shaded) area enclosed by the dispersion function in the region of the bending magnets (off-axis quadrupoles) illustrates the value and sign of $R_{56}$ achieved.

The dogleg-chicane section (S2) can produce chicane-like, neutral and dogleg-like values of $R_{56}$. Set-ups that achieve values of 0.20 mm, 0.00 mm and − 0.22 mm are shown in Fig. 5.

The set-up for $R_{56}={0.00}\,\text{mm}$ was considered first, and the result is shown in Fig. 5b. A symmetric solution is achieved that closes the dispersion function at the end of the section (S2) and controls the beta-functions to $\beta _{x,y}<{30}\,\text {m}$. The maximum quadrupole strength required for this set-up is $k={15.38}\,\text {m}^{-2}$, which is within the quadrupole design parameters. The offset quadrupoles (S2QD) require strengths of $k={-8.58}\,\text {m}^{-2}$, resulting in a beam pipe offset inside these quadrupoles of 4.1 mm. To accommodate this, the offset quadrupoles require a larger inscribed diameter of 27 mm, which reduces their maximum field strength to $|k|\simeq {12}\,\text {m}^{-2}$. Therefore the dogleg-chicane section of the beamline requires the use of two quadrupole designs: seven on-axis quadrupoles with an inscribed diameter of 21 mm and maximum field strength of $|k|\simeq {22.5}\,\text {m}^{-2}$, and four offset quadrupoles with an inscribed diameter of 27 mm and maximum field strength of $|k|\simeq {12}\,\text {m}^{-2}$.

The maximum chicane-like value of $R_{56}$ is found to be 0.20 mm and this result is shown in Fig. 5a. The positive value of $R_{56}$ returned by MAD8 is visually verified by the shaded areas that lie above the *s*-axis in the dispersion plot of Fig. 5a. This result is at the limit of acceptable maximum values of the beta-functions (as $\beta _{y}\gtrsim {100}\,\text {m}$) and quadrupole strengths, as it requires quadrupoles S2Q1 and S2Q7 to have the maximum design strength of $k={22.5}\,\text {m}^{-2}$.

The maximum dogleg-like value of $R_{56}$ achieved is − 0.22 mm and this result is shown in Fig. 5c. The negative value of achieved $R_{56}$ is visually verified by the shaded areas below the *s*-axis in the dispersion plot of Fig. 5c. This result is limited by the maximum value of the beta-functions; requesting more negative values of $R_{56}$ in MAD8 returned solutions with maximum beta-function values over 1000 m. For the maximum dogleg-like $R_{56}$ case, the required quadrupole strengths are within the design limits, with a maximum required strength of $k={15.35}\,\text {m}^{-2}$.

#### Full beamline (S1 S2 S3)

**Figure 6:**
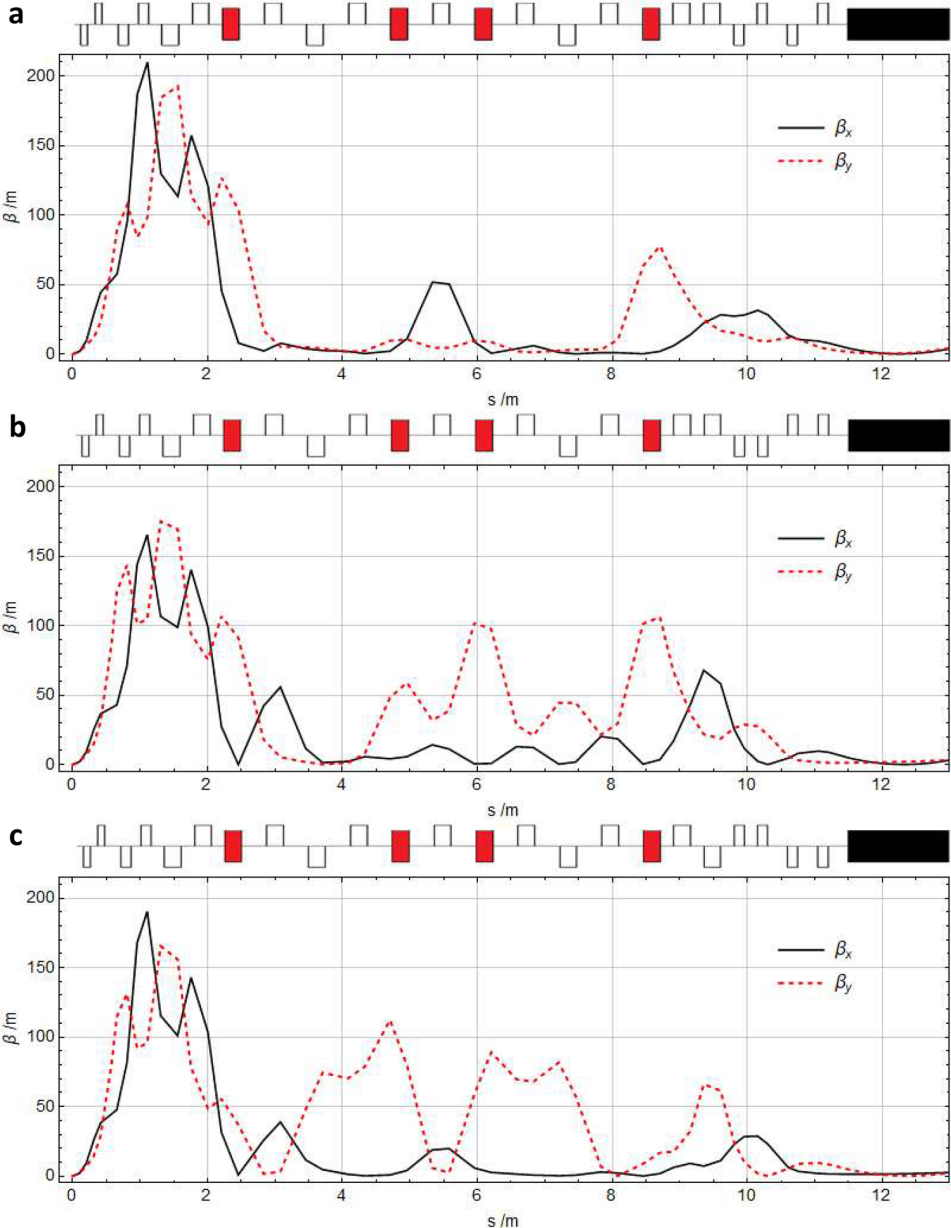
Magnet configurations and beta-functions for the whole beamline (sections S1, S2, S3 and the undulator) for the three set-ups of $R_{56}$. (**a**) $R_{56}={0.20}\,\text{mm}$ (chicane-like), (**b**) $R_{56}={0.00}\,\text{mm}$ (neutral) and (**c**) $R_{56}={-0.22}\,\text{mm}$ (dogleg-like). The magnet configuration is shown above each plot, which includes: focusing quadrupoles (upper empty rectangles); defocusing quadrupoles (lower empty rectangles); offset defocusing quadrupoles (red filled rectangles) and the undulator (black filled rectangle). The magnet strengths vary between each $R_{56}$ set-up. However, the configuration (order of focusing and defocusing quadrupoles) only varies in the final section of the beamline (S3); between the last offset quadrupole and the undulator. Each set-up produces maximum beta-function values in the focusing section (S1) and small values through the undulator. The beam transport length from the plasma exit to the undulator entrance (sections S1, S2 and S3) is 11.5 m.

The focusing (S1) and undulator matching (S3) sections are successfully configured to match each $R_{56}$ set-up of the dogleg-chicane section (S2) to the start and end conditions (Twiss parameters) of the beamline. The resulting three set-ups of the full beamline are shown in Fig. 6.

The focusing section (S1) is matched with a preference for larger values of the beta-functions occurring close to the plasma exit to reduce the resulting beam size throughout the transport. As the emittance of an LWFA electron beam increases with distance along a beamline, it follows that the beam size represented by a given beta-function value will also increase along the beamline. Therefore, to reduce the beam size at all points, it is preferable to allow larger beta-function values to occur close to the plasma source rather than further downstream. In all three beamline set-ups, the maximum beta-function value is limited to $\beta _{x,y}\lesssim {200}\,\text {m}$. Matching the focusing section (S1) for the case of $R_{56}={0.00}\,\text{mm}$ produces a configuration with the first PMQ defocusing and the second PMQ focusing. As the PMQs cannot easily be changed from their configuration (this would require physically swapping their positions or rotating each PMQ by ${90}^{\circ }$), this FODO arrangement is maintained for the chicane-like and dogleg-like solutions of $R_{56}$.

In contrast, in the undulator matching section (S3), the quadrupole configuration is allowed to vary between each $R_{56}$ set-up. This variability is practical, as all the quadrupoles in this section (S3) are electromagnetic and can easily be changed from focusing to defocusing by changing the direction of current through the magnets’ coils. This added flexibility enables all three set-ups of $R_{56}$ to be successfully matched to the undulator. The different configurations of the magnets in the undulator matching section are compared in Fig. 6a–c. In each case, the undulator matching section reduces the beta-functions to $\beta _{x,y}<{5}\,\text {m}$ throughout the length of the undulator.

The final three full beamline set-ups achieve $R_{56}$ values of 0.20 mm, 0.00 mm and − 0.22 mm while matching the dogleg-chicane section with the start and end conditions. The total length of the full beamline, from plasma exit to undulator entrance is 11.5 m, which is within the design limit of 13.9 m. The magnet strengths are also within the design limits; $|k|<{12}\,\text {m}^{-2}$ for the offset quadrupoles, $|k|\le {22.5}\,\text {m}^{-2}$ for all other electromagnetic quadrupoles and ${7.5}\,\text {m}^{-2}\le |k|\le {29}\,\text {m}^{-2}$ for the permanent magnet quadrupoles. These final three full beamline set-ups could, therefore, be practically implemented at the SCAPA facility^42^.

### Tracked particles

**Figure 7:**
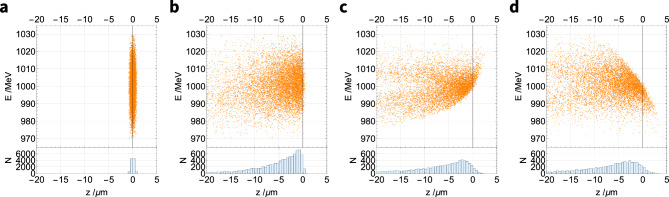
The longitudinal phase space of a 1.0 GeV electron bunch with no initial chirp (**a**) before and (**b**–**d**) after it travels through the three beamline set-ups with differing values of $R_{56}$. (**a**) The bunch has an initial length of 0.3 $\upmu \text {m}$ (1 fs). (**b**) After travelling through the 11.5 m beamline with $R_{56}={0.00}\,\text{mm}$ the bunch length has increased to 10 $\upmu \text {m}$ (34 fs) at the beamline end (undulator entrance) because of divergence-driven lengthening. (**c**) The chicane-like set-up ($R_{56}={0.20}\,\text{mm}$) causes the bunch to develop a positive chirp. (**d**) The dogleg-like set-up ($R_{56}=-{0.22}\,\text{mm}$) causes the bunch to develop a negative chirp. All plots use the same scale and bunch lengths values are root-mean-squared (r.m.s.).

Three electron bunches with zero, negative and positive chirp have been tracked through the three $R_{56}$ set-ups of the beamline to demonstrate their effect on the bunch chirp. The results for an initially upright (zero chirp) bunch are shown in Fig. 7. At the start of the beamline (plasma exit), the bunch has an r.m.s. length of 0.3 μm (1.0 fs) as shown in Fig. 7a, which naturally lengthens as the bunch travels to an r.m.s. length of 10 $\upmu \text {m}$ (34 fs) at the beamline end (undulator entrance), as shown in Fig. 7b. This bunch lengthening is caused by the relatively large divergence of the beam; electrons with initially high transverse momenta take a longer (outer) path through the quadrupoles and fall behind the centre of the bunch^44^. The divergence-driven bunch lengthening affects the final bunch length in all three beamline set-ups, as shown in Fig. 7b–d. Fig. 7c shows that tracking the same initial bunch through the chicane-like $R_{56}$ set-up produces the expected behaviour: the high-energy part (top) of the bunch is sheared forward, resulting in a positive chirp. Similarly, the dogleg-like $R_{56}$ set-up causes the top of the bunch to shear backward, producing a negative chirp as shown in Fig. 7d. This demonstrates the unique feature of this beamline: a bunch can be sheared in either direction in longitudinal phase space to apply a positive or negative chirp to the bunch.

**Figure 8:**
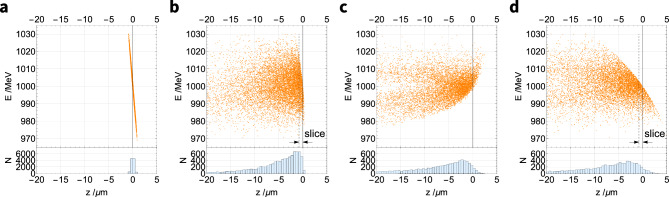
The longitudinal phase space of a 1.0 GeV electron bunch with initially negative chirp (**a**) before and (**b**–**d**) after it travels through the three beamline set-ups with differing values of $R_{56}$. (**a**) The bunch has an initial length of 0.3 $\upmu \text {m}$ (1.0 fs) and a negative chirp. (**b**) After travelling through the 11.5 m beamline with $R_{56}={0.00}\,\text{mm}$ the bunch length increases to 10 $\upmu \text {m}$ (34 fs) at the beamline end (undulator entrance) because of divergence-driven lengthening. (**c**) The chicane-like set-up ($R_{56}={0.20}\,\text{mm}$) shears the bunch beyond a vertical position to produce a positive chirp. (**d**) The dogleg-like set-up ($R_{56}=-{0.22}\, \text{mm}$) enhances the negative chirp of the bunch. A coherent slice of the bunch has a width equal to the coherence length (0.8 $\upmu \text {m}$) and is shown in (**b**) and (**d**). The energy spread of a coherent slice is greater for a more upright bunch (**b**) than for a chirped bunch (**d**). All plots use the same scale and bunch lengths values are root-mean-squared (r.m.s.).

Figure 8 shows the effect of the three beamline set-ups on a negatively chirped bunch. As the initial bunch length and total energy spread are the same as for the upright (zero chirp) bunch, the chirp only appears as a small tilt in Fig. 8a. Figure 8b shows the effect of transport through the $R_{56}={0.00}\,\text {mm}$ beamline set-up; the initial negative chirp is maintained and the same lengthening (10 $\upmu \text {m}$) of the bunch occurs as in Fig. 7b, caused by path length differences between particles of different transverse momenta. Fig. 8c shows the effect of the chicane-like set-up (with $R_{56}={0.20}\,\text {mm}$) on the bunch with an initially negative chirp. The chicane-like set-up overcompensates for the negative chirp, shearing the bunch beyond the upright position, resulting in a bunch with a positive chirp. This set-up uses the maximum chicane-like value of $R_{56}$; a smaller magnitude value of $R_{56}$ can be produced to shear the bunch to an upright position, which shortens the bunch compared with the $R_{56}={0.00}\;\text {mm}$ case. This technique to shorten the bunches by compensating for their initial chirp is most effective with longer initial bunch lengths, which have an increased potential for shortening. Shortening a bunch can promote coherent radiation at wavelengths longer than the bunch length, which for a 1 fs bunch (bunch length 300 nm) would be in the ultraviolet range^58^ and for a 34 fs bunch (10,000 nm) would be in the infrared range. Shortening a chirped bunch also increases the peak current of the bunch, which is beneficial for the production of free-electron laser (FEL) radiation.

Figure 8d shows the effect of the dogleg-like $R_{56}$ set-up of the beamline (with $R_{56}=-{0.22}\,\text {mm}$) on a bunch with an initial negative chirp. As expected, this causes the top of the bunch to shear backward, enhancing the negative chirp and lengthening the bunch. The bunch length increases to r.m.s 37 $\upmu \text {m}$ (123 fs), which is around four times the length caused by the $R_{56}={0.00}\,\text {mm}$ set-up. Lengthening the bunch in this way reduces the slice emittance and so promotes the production of FEL radiation in an undulator^40^. The length of a bunch slice that radiates coherently in an FEL is given by the coherence length $L_{coh}$,

2 $$\begin{aligned} L_{coh}\simeq \frac{\lambda _{r}}{2\rho _{P}\sqrt{\pi }}, \end{aligned}$$

where $\lambda _{r}$ is the radiation wavelength and $\rho _{P}$ is the dimensionless FEL Pierce parameter^59^. The fundamental radiation wavelength for a 1.0 GeV bunch travelling through the ALPHA-X undulator is $\lambda _{r}={2.8}\,\text {nm}$ and a typical value of the Pierce parameter for short-wavelength FELs is^60^ $\rho _{P}\approx 10^{-3}$. Consequently, the longitudinal length of a coherent slice of the bunch used in this study is around $L_{coh}\approx {0.8}\upmu \text {m}$ and this length is indicated in Fig. 8b,d. The slice indicated in Fig. 8b contains particles with a range of energies, approximately equal to the total energy spread of the bunch (${1}{\%}$), whereas an equivalent slice shown in Fig. 8d contains particles with a smaller range of energies, around ${0.3}{\%}$. Transverse emittance growth of the LWFA bunch along a beamline is a chromatic effect; a transverse phase-space ellipse will rotate as it passes along a FODO channel and this rotation occurs at different rates for different energies. The slice shown in Fig. 8b contains a large (1%) energy spread and so will experience the chromatic effect of transverse emittance growth: the emittance of this slice is similar to the total bunch emittance. In contrast, the bunch slice shown in Fig. 8d has a smaller energy spread (0.3%), so will contain fewer overlapping transverse phase-space ellipses and experience less emittance growth during transport. Lengthening the bunch as shown in Fig. 8d can therefore reduce the slice emittance to promote FEL radiation production in an undulator.

Particle tracking has also been carried out for a bunch with an initially positive chirp, which yields similar results to those discussed above. In all cases, some divergence-driven bunch length growth is caused by particles with high transverse momenta taking a longer path. This issue can be reduced by adding collimators in regions where the bunch size is large, thereby removing these particles from the bunch and reducing the bunch tails in Figs. 7 and 8. The addition of collimators to further reduce the bunch length may allow the whole bunch to radiate coherently in the undulator (CSR). Collimation also reduces the total bunch charge and therefore the achievable peak current, which may be detrimental to FEL radiation production. Further study into the effect of collimation on the production of radiation in the two schemes is necessary to determine whether this would bring an overall benefit to the system.

## Discussion

The beamline presented here can produce both positive and negative values of longitudinal dispersion ($R_{56}$). Few other beamlines have been designed with this capability; one example is an arc design for the MAX-IV linear accelerator^61^. Although the MAX-IV arc can produce a larger range of $R_{56}$ variation, its design follows a curved path. The beamline presented here is unique, as the beam returns to the same straight-line axis and is the first such beamline designed for use with an LWFA source.

MAD8 optics and particle tracking simulations show that our beamline can successfully transport electrons from a laser wakefield accelerator to an undulator. The beamline incorporates both adjustable-strength permanent magnet quadrupoles and normal conducting quadrupoles, which focus the beam, adjust the $R_{56}$ and match the beam to an undulator. The design includes a novel adjustable dogleg-chicane section (S2), which can vary the $R_{56}$ from a chicane-like value of 0.20 mm to a dogleg-like value of − 0.22 mm. A neutral 0.00 mm solution is also demonstrated, which suggests that further solutions can be found within the given range. The $R_{56}$ variation allows the chirped nature of laser wakefield electrons to be utilised. We demonstrate that bunches can be sheared forward or backward, imparting either a negative or positive chirp onto the electron bunch.

Chirp adjustment of electron bunches from LWFAs has the potential to enhance the radiation produced in a downstream undulator. Adjusting the chirp to lengthen the bunch can reduce the slice emittance, potentially promoting FEL radiation. Alternatively, shortening an initially chirped bunch may promote FEL radiation because of the increased peak current. The effect of shortening or lengthening these bunches on the radiation produced in an undulator requires further investigation and can be explored experimentally with this adjustable beamline design. LWFA bunches naturally lengthen during beam transport due to their relatively large initial divergence as particles with larger transverse momenta take a longer outer path through the quadrupoles. Future studies to remove these divergent particles, for example by collimation, would additionally contribute to controlling the bunch length of transported LWFA electron bunches.

In summary, we present a practical design for a flexible experimental beamline that can adjust the chirp of electron bunches produced by a laser wakefield accelerator and transport these bunches to an undulator. The proposed beamline allows various experiments driven by LWFA sources to be carried out, including measurements of the chirp orientation of LWFA-produced electron bunches and exploring the effect of bunch chirp on the radiation produced in a downstream undulator. The investigations made possible by this beamline would benefit the development of a future LWFA-driven FEL.

## Data Availability

The data supporting the findings reported in this paper are openly available from the Zenodo repository at http://doi.org/10.5281/zenodo.7022650.

## References

[1] Leemans WP (2006). GeV electron beams from a centimetre-scale accelerator. Nat. Phys..

[2] Wang X (2013). Quasi-monoenergetic laser-plasma acceleration of electrons to 2 GeV. Nat. Commun..

[3] Leemans W (2014). Multi-GeV electron beams from capillary-discharge-guided subpetawatt laser pulses in the self-trapping regime. Phys. Rev. Lett..

[4] Gonsalves A (2019). Petawatt laser guiding and electron beam acceleration to 8 GeV in a laser-heated capillary discharge waveguide. Phys. Rev. Lett..

[5] Weingartner R (2012). Ultralow emittance electron beams from a laser-wakefield accelerator. Phys. Rev. ST Accel. Beams.

[6] Kneip S (2012). Characterization of transverse beam emittance of electrons from a laser-plasma wakefield accelerator in the bubble regime using betatron x-ray radiation. Phys. Rev. ST Accel. Beams.

[7] Qin Z (2018). Ultralow-emittance measurement of high-quality electron beams from a laser wakefield accelerator. Phys. Plasmas.

[8] Rechatin C (2010). Characterization of the beam loading effects in a laser plasma accelerator. New J. Phys..

[9] Lundh O (2011). Few femtosecond, few kiloampere electron bunch produced by a laser-plasma accelerator. Nat. Phys..

[10] Buck A (2011). Real-time observation of laser-driven electron acceleration. Nat. Phys..

[11] Islam MR (2015). Near-threshold electron injection in the laser-plasma wakefield accelerator leading to femtosecond bunches. New J. Phys..

[12] Zhang C (2016). Temporal characterization of ultrashort linearly chirped electron bunches generated from a laser wakefield accelerator. Phys. Rev. Accel. Beams.

[13] Jaroszynski DA, Vieux G (2002). Coherent radiation sources based on laser plasma accelerators. AIP Conf. Proc..

[14] Rossbach J, Schneider JR, Wurth W (2019). 10 years of pioneering X-ray science at the free-electron laser FLASH at DESY. Phys. Rep..

[15] Floettmann K (2003). Some basic features of the beam emittance. Phys. Rev. Spec. Top. Accel. Beams.

[16] Floettmann K (2003). Erratum: Some basic features of the beam emittance. Phys. Rev. Spec. Top. Accel. Beams.

[17] Antici P (2012). Laser-driven electron beamlines generated by coupling laser-plasma sources with conventional transport systems. J. Appl. Phys..

[18] Li X, Chancé A, Nghiem PAP (2019). Preserving emittance by matching out and matching in plasma wakefield acceleration stage. Phys. Rev. Accel. Beams.

[19] Migliorati M (2013). Intrinsic normalized emittance growth in laser-driven electron accelerators. Phys. Rev. ST Accel. Beams.

[20] McGuffey C (2010). Ionization induced trapping in a laser wakefield accelerator. Phys. Rev. Lett..

[21] Couperus JP (2017). Demonstration of a beam loaded nanocoulomb-class laser wakefield accelerator. Nat. Commun..

[22] Dornmair I, Floettmann K, Maier A (2015). Emittance conservation by tailored focusing profiles in a plasma accelerator. Phys. Rev. ST Accel. Beams.

[23] Barber S (2017). Measured emittance dependence on the injection method in laser plasma accelerators. Phys. Rev. Lett..

[24] Hu R (2016). Brilliant GeV electron beam with narrow energy spread generated by a laser plasma accelerator. Phys. Rev. Accel. Beams.

[25] Zhang Z (2015). Generation of high quality electron beams from a quasi-phase-stable cascaded laser wakefield accelerator with density-tailored plasma segments. New J. Phys..

[26] Wang W (2016). High-brightness high-energy electron beams from a laser wakefield accelerator via energy chirp control. Phys. Rev. Lett..

[27] Manahan GG (2017). Single-stage plasma-based correlated energy spread compensation for ultrahigh 6D brightness electron beams. Nat. Commun..

[28] Zhang Z (2016). Energy spread minimization in a cascaded laser wakefield accelerator via velocity bunching. Phys. Plasmas.

[29] Döpp A (2018). Energy-chirp compensation in a laser wakefield accelerator. Phys. Rev. Lett..

[30] Schlenvoigt H-P (2008). A compact synchrotron radiation source driven by a laser-plasma wakefield accelerator. Nat. Phys..

[31] Anania MP (2014). An ultrashort pulse ultra-violet radiation undulator source driven by a laser plasma wakefield accelerator. Appl. Phys. Lett..

[32] Wiggins, S. M. et al. Undulator radiation driven by laser-wakefield accelerator electron beams. In *Proceedings of SPIE Optics and Optoelectronics 2015*, vol. 9509, 8, 10.1117/12.2178847 (International Society for Optics and Photonics, 2015).

[33] Fuchs M (2009). Laser-driven soft-X-ray undulator source. Nat. Phys..

[34] Wang W (2021). Free-electron lasing at 27 nanometres based on a laser wakefield accelerator. Nature.

[35] Liu T, Zhang T, Wang D, Huang Z (2017). Compact beam transport system for free-electron lasers driven by a laser plasma accelerator. Phys. Rev. Accel. Beams.

[36] Bernhard A (2018). Progress on experiments towards LWFA-driven transverse gradient undulator-based FELs. Nucl. Instrum. Methods Phys. Res. Sect. A Accel. Spectrom. Detect. Assoc. Equip..

[37] Huang Z, Ding Y, Schroeder CB (2012). Compact X-ray free-electron laser from a laser-plasma accelerator using a transverse-gradient undulator. Phys. Rev. Lett..

[38] Seggebrock T, Maier AR, Dornmair I, Grüner F (2013). Bunch decompression for laser-plasma driven free-electron laser demonstration schemes. Phys. Rev. ST Accel. Beams.

[39] Couprie M-E (2015). Towards the next generation of short wavelength light sources. J. Phys. Conf. Ser..

[40] Maier AR (2012). Demonstration scheme for a laser-plasma-driven free-electron laser. Phys. Rev. X.

[41] André T (2018). Control of laser plasma accelerated electrons for light sources. Nat. Commun..

[42] Wiggins, S. M. *et al.* Application programmes at the Scottish Centre for the Application of Plasma-based Accelerators (SCAPA). In *Relativistic Plasma Waves and Particle Beams as Coherent and Incoherent Radiation Sources III*, vol. 11036, 110360T, 10.1117/12.2520717 (International Society for Optics and Photonics, 2019).

[43] Shepherd BJ, Clarke JA (2011). Design, measurement and correction of a pair of novel focusing undulators for the ALPHA-X project. Nucl. Instrum. Methods Phys. Res. Sect. A Accel. Spectrom. Detect. Assoc. Equip..

[44] Dewhurst, K. A. Transport of electrons from a laser wakefield accelerator to produce short-wavelength radiation in undulators. Ph.D. thesis, The University of Manchester, Manchester, UK (2021).

[45] Yu C (2016). Ultrahigh brilliance quasi-monochromatic MeV $$\gamma$$-rays based on self-synchronized all-optical Compton scattering. Sci. Rep..

[46] Pollock BB (2011). Demonstration of a narrow energy spread, 0.5 GeV electron beam from a two-stage laser wakefield accelerator. Phys. Rev. Lett..

[47] Tooley M (2017). Towards attosecond high-energy electron bunches: Controlling self-injection in laser-wakefield accelerators through plasma-density modulation. Phys. Rev. Lett..

[48] Golovin G (2016). Intrinsic beam emittance of laser-accelerated electrons measured by x-ray spectroscopic imaging. Sci. Rep..

[49] Chen S (2013). MeV-energy X rays from inverse Compton scattering with laser-wakefield accelerated electrons. Phys. Rev. Lett..

[50] Grote, H. & Iselin, F. C. The MAD Program (Methodical Accelerator Design) User’s Reference Manual (1994).

[51] Manahan GG (2014). Characterization of laser-driven single and double electron bunches with a permanent magnet quadrupole triplet and pepper-pot mask. New J. Phys..

[52] Shepherd BJA (2014). Tunable high-gradient permanent magnet quadrupoles. J. Inst..

[53] Shepherd, B. J. A. Development of PM-based Quadrupole Magnet Prototypes for the CLIC Drive Beam (2019). CLIC Workshop Talk.

[54] Constantinides S, Croat J, Ormerod J (2022). Chapter 11—Permanent magnet coatings and testing procedures. Modern Permanent Magnets, Woodhead Publishing Series in Electronic and Optical Materials.

[55] Machida S (2012). Acceleration in the linear non-scaling fixed-field alternating-gradient accelerator EMMA. Nat. Phys..

[56] Borland M (2001). Simple method for particle tracking with coherent synchrotron radiation. Phys. Rev. Spec. Top. Accel. Beams.

[57] Floettmann, K. ASTRA A Space Charge Tracking Algorithm (2017).

[58] Brunetti E (2021). Vacuum ultraviolet coherent undulator radiation from attosecond electron bunches. Sci. Rep..

[59] Bonifacio R, Casagrande F (1985). The superradiant regime of a free electron laser. Nucl. Instrum. Methods Phys. Res. Sect. A Accel. Spectrom. Detect. Assoc. Equip..

[60] Chao AW, Mess KH, Tigner M, Zimmermann F (2013). Handbook of Accelerator Physics and Engineering.

[61] Williams PH (2020). Arclike variable bunch compressors. Phys. Rev. Accel. Beams.

